# Sorcin Inhibits Mitochondrial Apoptosis by Interacting with STAT3 via NF-κB Pathway

**DOI:** 10.3390/ijms25137206

**Published:** 2024-06-29

**Authors:** Yizi Li, Manlin Tian, Jaceline Gislaine Pires Sanches, Qingqing Zhang, Li Hou, Jun Zhang

**Affiliations:** 1 Department of Pathology and Forensic Medicine, College of Basic Medical Sciences, Dalian Medical University, Dalian 116044, China; 17304807750@163.com (Y.L.); tianmanlin0214@163.com (M.T.); jacelinesanches26@gmail.com (J.G.P.S.); zqqzfg@163.com (Q.Z.); lihou1972@163.com (L.H.); 2 State Key Laboratory of Oncology in South China, Sun Yat-sen University, Guangzhou 510275, China

**Keywords:** STAT3, SRI, apoptosis, protein–protein interaction

## Abstract

Hepatocellular carcinoma (HCC) is a common tumor. Our group has previously reported that sorcin (SRI) plays an important role in the progression and prognosis of HCC. This study aims to explore the mechanism of SRI inhibiting the mitochondrial apoptosis. Bioinformatics analysis, co-IP and immunofluorescence were used to analyze the relationship between SRI and STAT3. MMP and Hoechst staining were performed to detect the effect of SRI on cell apoptosis. The expression of apoptosis-related proteins and NF-κB signaling pathway were examined by Western blot and immunohistochemistry when SRI overexpression or underexpression in vivo and in vitro were found. Moreover, inhibitors were used to further explore the molecular mechanism. Overexpression of SRI inhibited cell apoptosis, which was attenuated by SRI knockdown in vitro and in vivo. Moreover, we identified that STAT3 is an SRI-interacting protein. Mechanistically, SRI interacts with STAT3 and then activates the NF-κB signaling pathway in vitro and in vivo. SRI interacting with STAT3 inhibits apoptosis by the NF-κB pathway and further contributes to the proliferation in HCC, which offers a novel clue and a new potential therapeutic target for HCC.

## 1. Introduction

Hepatocellular carcinoma (HCC) is the sixth most common cancer and the fourth leading cause of cancer-related mortality worldwide [1,2]. Although the treatments have greatly improved over the past several decades, HCC is still a serious threat to public health [3,4]. However, the molecular mechanisms of HCC are not fully understood [5]. Therefore, exploring the molecular mechanism of HCC growth, progression and identifying new treatment targets is still eagerly needed.

Apoptosis resistance or evasion is one of the hallmarks of cancer. It has been acknowledged that inhibition of apoptosis is associated with metastasis [6]. Apoptotic cell death is mediated by either the intrinsic or the extrinsic apoptotic pathway [7], which are controlled by pro- and anti-apoptotic proteins of the Bcl-2 family. The Bcl-2 family proteins (Bcl-2, Bcl-XL, Bcl-W and MCL-1) prevent uncontrolled initiation of apoptosis, while pro-apoptotic Bcl-2 proteins (Bak, Bax, and Bok) trigger mitochondrial outer membrane permeability (MOMP) [8]. The modulation of Bcl-2 family proteins results in dissipation of MMP and the subsequent release of many proteins to promote apoptosis [9]. Therefore, it is paramount to explore the molecular mechanism of HCC cell apoptosis so as to provide a hopeful target for HCC therapy.

Soluble resistance-related calcium-binding protein (sorcin, SRI) is a 22-kDa, soluble, small, penta EF family (PEF) of calcium (Ca^2+^)-binding protein which has an association with cancer development [10]. SRI was found to be overexpressed in different cancers, including breast cancer, colorectal cancer, gastric cancer, leukemia, lung cancer, nasopharyngeal cancer and ovarian cancer [11]. Previous reports revealed that SRI was involved in protecting against mitochondrial apoptosis and was relevant to anti-apoptotic activities in tumor cells [12,13,14,15]. However, the exact mechanisms of its action in the initiation and progression of HCC remain doubtful.

Signal transducers and activators of transcription (STATs) are a vital protein family of transcription factors widely studied in cancer [16]. STAT3, one of the seven members of the STAT family, is constitutively activated in a variety of tumors, including HCC [17,18,19,20,21], and plays a key role in several biological processes such as resisting apoptosis and proliferation [22]. Growing evidence demonstrates that STAT3 plays an important role in HCC development, progression, and prognosis [23]. STAT3 usually resides in the cytoplasm, but activated STAT3 can start the transcription of its target genes, including Bcl-2, Bcl-XL and MCL-1 [24,25]. Inhibition of constitutively STAT3 activation can induce apoptosis and inhibit cancer cell growth, indicating STAT3 is required for cancer cell survival and growth [17,26,27,28]. It was reported that SRI interacted with STAT3 in hepatic inflammation [29]. Moreover, the cooperation between STAT3 and NF-κB may also occur to promote the progression of HCC [30], but the specific mechanism is not well defined at present.

The nuclear factor-κB (NF-κB) signaling pathway plays a crucial role in tumorigenesis [31]. Persistent activation of NF-κB signaling inhibits apoptosis [32]. NF-κB comprises five family members, and p65 is particularly significant to NF-κB activity because p65 is a primary subunit of the NF-κB complex expressed in almost all tissues [33]. NF-κB exerts its anti-apoptotic function through the transcription of anti-apoptotic proteins, which can be divided into two groups. One is the inhibitor of apoptosis proteins (IAPs), cIAP1, cIAP2, and the other is Bcl-2 family members, including Bcl-2, Bcl-XL and MCL-1 [34]. Thus, backed by available evidence, it stands to reason that NF-κB signaling plays an essential role in HCC apoptosis.

In the current study, we found SRI is overexpressed in HCC patients and closely associated with various clinicopathological features. Moreover, we demonstrated that SRI interacts with STAT3, selectively targeting NF-κB signaling pathway, mediating apoptosis in vitro and in vivo. Thus, SRI might be a potential prognostic biomarker and a valuable therapeutic target for HCC.

## 2. Results

### 2.1. SRI and STAT3 Are Overexpressed and Closely Correlated in HCC Tissues and Cells

**Figure 1:**
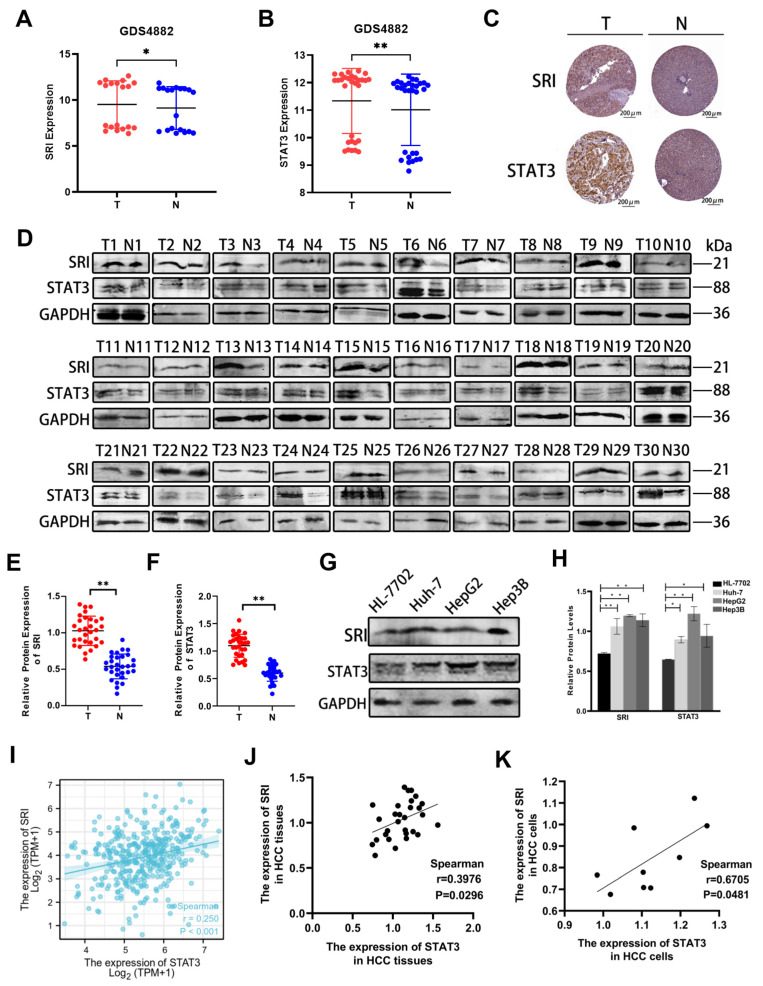
SRI and STAT3 are upregulated both in HCC tissues and cells. (**A**,**B**) SRI and STAT3 are highly expressed in HCC tissues (T) compared with normal liver tissues (N) in the GEO database (GDS4882). (**C**) Image available from Proteinatlas database showed high expression of SRI and STAT3 in tumor (T) compared with normal liver tissues (N). The expression of proteins were determined by the brown area. Scale bars = 200 μm. (**D**–**H**) Western blot showed SRI and STAT3 proteins were overexpressed in clinical HCC tissues and in Huh-7/HepG2/Hep3B cells. The red and blue spots represented the expression of different proteins in the T and N groups, respectively. (**I**) From the TGGA database, SRI was found to be positively correlated with STAT3 in LIHC (*p* < 0.001). (**J**,**K**) The expressions of SRI and STAT3 are positively correlated in HCC tissues and cells lines (*p* < 0.05). * *p* < 0.05, ** *p* < 0.01.

HCC samples and normal liver samples were acquired from the dataset of GEO. As shown in Figure 1A,B, SRI expression was high in HCC samples compared to normal liver samples and STAT3 expression was also high in HCC samples. The image available from the Proteinatlas database are also consistent with GEO (Figure 1C). To further confirm SRI and STAT3 expression, we collected clinical samples of HCC and adjacent non-tumor tissues to perform Western blot. Western blot results showed that SRI and STAT3 expression were significantly increased in HCC specimens (Figure 1D–F). Furtherly, in HCC cell lines (Huh-7, HepG2, and Hep-3B cells), SRI and STAT3 were also overexpressed (Figure 1G,H). Altogether, SRI and STAT3 are highly expressed both in HCC tissues and cells.

Particularly, RNAseq data of HCC patients obtained from the TCGA dataset showed that SRI expression correlated positively with STAT3 (*p* < 0.001) (Figure 1I), which was further confirmed in HCC cells and tissues (Figure 1J,K). Altogether, the expressions of SRI and STAT3 are not only strongly high, but also closely correlated with each other.

Furthermore, the TGGA database was also queried to verify whether SRI protein expression was significantly correlated with clinical T stage (*p* = 0.013), pathologic stage (*p* = 0.025), tumor status (*p* = 0.018) and OS event (*p* = 0.042) of HCC patients (Table 1), indicating that SRI takes part in the development and progression of HCC.

### 2.2. Apoptosis-Related Proteins Take Part in HCC

**Figure 2:**
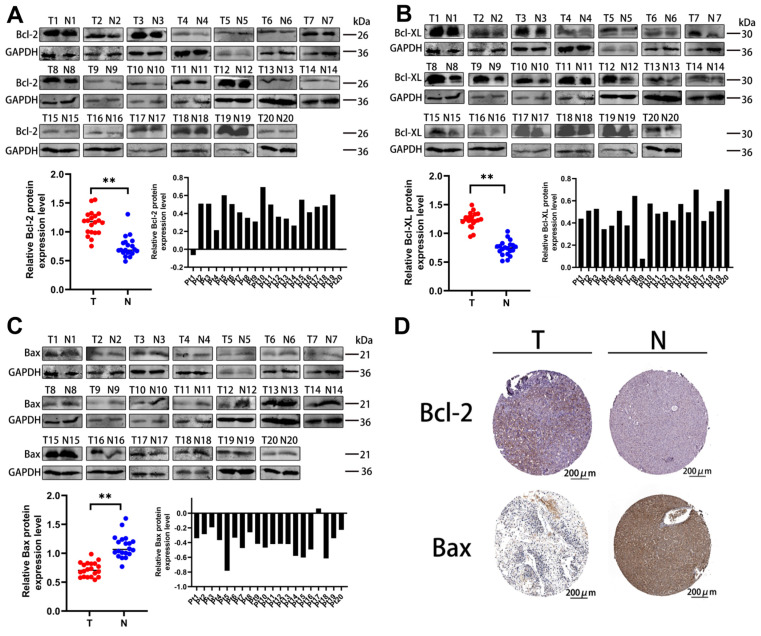
Anti-apoptosis-related proteins are highly expressed, while pro-apoptosis-related protein is lowly expressed in HCC tissues. (**A**,**B**) Expressions of Bcl−2 and Bcl−XL were increased in clinical HCC tissues. (**C**) Bax expression was decreased in clinical HCC tissues. The red and blue spots represented the expression of different proteins in the T and N groups, respectively. ** *p* < 0.01. (**D**) IHC staining images of Bcl−2 and Bax in HCC (T) and normal liver tissues (N) acquired from Proteinatlas database. The expression of proteins were determined by the brown area. Scale bars = 200 μm.

Interestingly, the Western blot results showed that the levels of Bcl-2 and Bcl-XL were significantly increased but Bax was decreased in HCC samples compared with normal liver samples (Figure 2A–C). At the same time, the IHC assay images from the Proteinatlas database are consistent with the above results (Figure 2D). Altogether, the high expression of anti-apoptosis-related proteins and the low expression of the pro-apoptosis-related protein are closely related to the development and progression of HCC.

### 2.3. SRI and STAT3 Are Interacting Proteins

**Figure 3:**
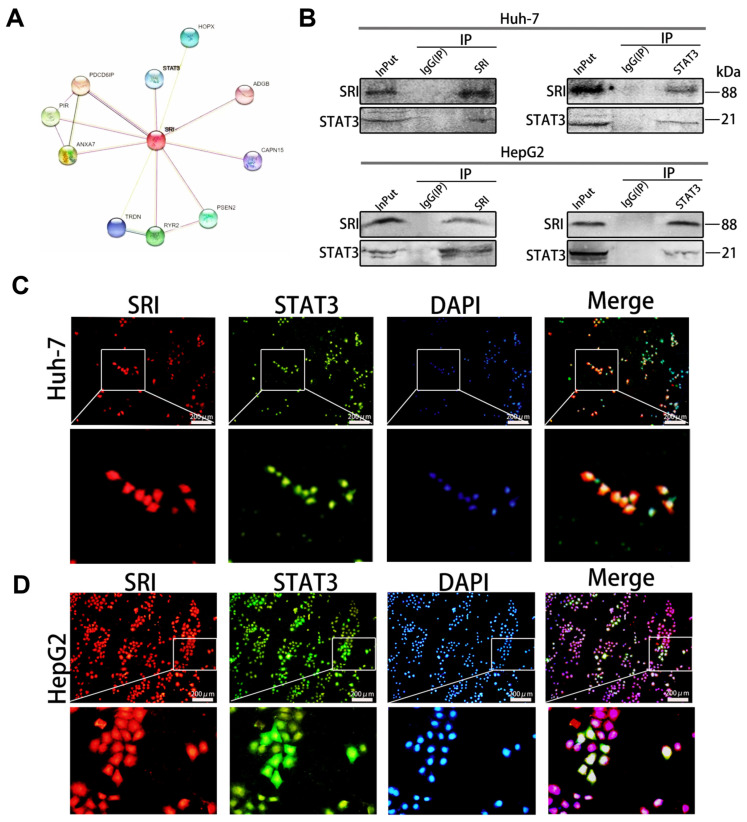
SRI and STAT3 are interacting proteins. (**A**) SRI and STAT3 are interacting proteins in the STRING database. (**B**) SRI and STAT3 are interacting proteins confirmed by co-immunoprecipitation assays in Huh-7/HepG2 cells. (**C**,**D**) SRI (shown in red) co-localized with STAT3 (shown in green) was visualized by cellular immunofluorescence in Huh-7/HepG2 cells. DAPI (shown in blue) was used for nuclear staining. Scale bars = 200 μm.

SRI and STAT3 are interacting proteins, as shown by STRING database (Figure 3A). Next, we confirmed the data by co-immunoprecipitation (Co-IP) and immunofluorescence staining experiments. The Co-IP results showed that endogenous STAT3 was coimmunoprecipitated with flag-tagged SRI and endogenous SRI was coimmunoprecipitated with STAT3 in Huh-7 and HepG2 cells (Figure 3B). Moreover, immunofluorescence staining confirmed that SRI and STAT3 co-localized mostly in Huh-7 and HepG2 cells (Figure 3C,D).

### 2.4. SRI Interacts with STAT3, Inhibits Apoptosis, and Activates the NF-κB Signaling Pathway In Vitro and In Vivo

**Figure 4:**
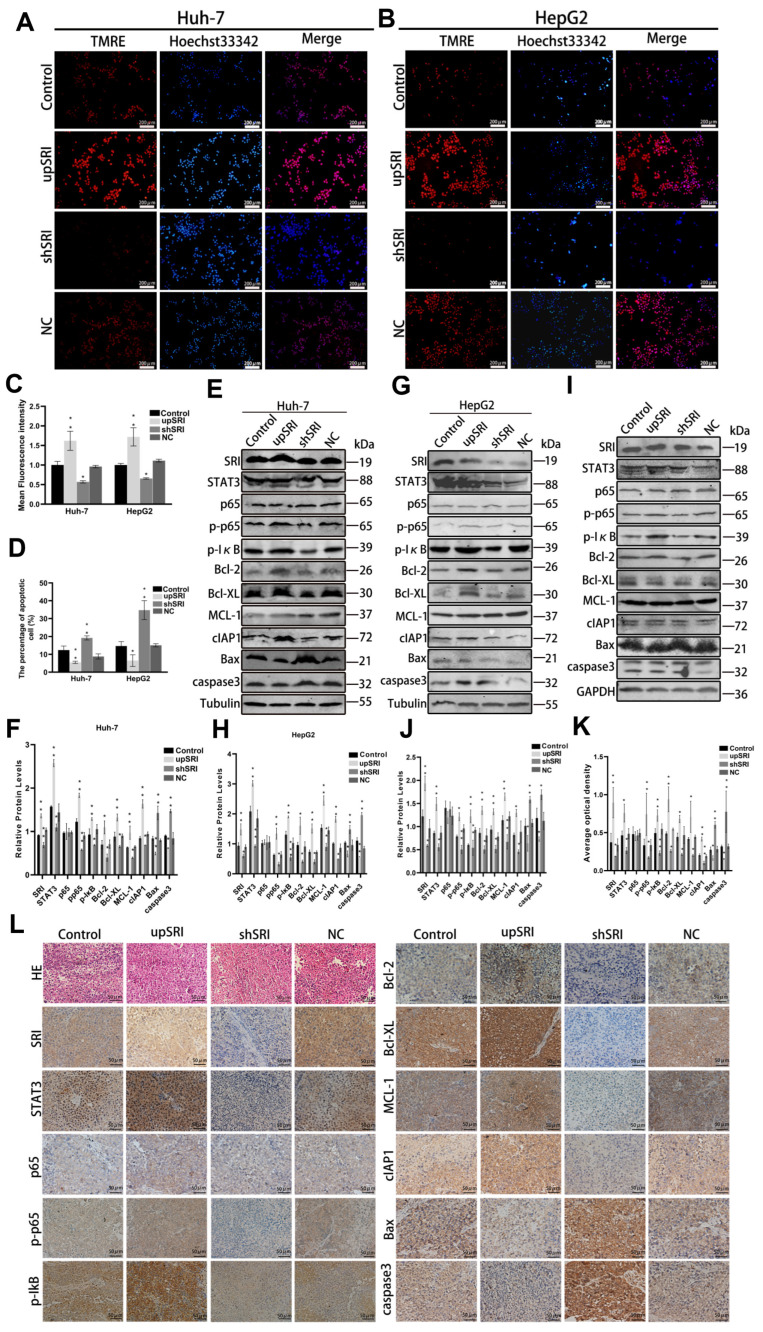
SRI interacts with STAT3, inhibits apoptosis and activates the NF-κB signaling pathway in vitro and in vivo. (**A**–**D**) SRI downexpression reduced the fluorescence intensity of TMRE (shown in red) and enhanced the apoptosis sensitivities by Hoechst 33342 (shown in blue) staining assay. The results of SRI overexpression were consisted with SRI downexpression. Scale bars = 200 μm. (**E**–**J**) Western blot detected the expression of STAT3, p65, p-p65, p-IκB and apoptosis-related proteins when SRI overexpression or downexpression were found in HCC cell and tumor xenografts. (**K**,**L**) Representative IHC images of STAT3, p65, p-p65, p-IκB and apoptosis-related proteins when SRI overexpression or downexpression were found in tumor xenografts. The expression of proteins were determined by the brown area. Scale bars = 50 μm. * *p* < 0.05, ** *p* < 0.01.

To detect the efficacy of SRI on cell apoptosis, the mitochondrial membrane potential (MMP) assay and Hoechst 33342 staining assay were performed. Compared with the control cells and NC cells, the fluorescence intensity of shSRI groups was reduced, while that of the upSRI groups was noticeably enhanced, as shown by the MMP assay (Figure 4A–D). The Hoechst 33342 staining assay showed that the number of apoptotic cells of shSRI groups with condensed and fragmented nuclei was higher, whereas that of the upSRI groups was lower. Collectively, these observations showed that SRI overexpression inhibits apoptosis, whereas SRI downexpression promotes apoptosis.

To further reveal the molecular mechanism of SRI on cell apoptosis in HCC, the Western blot assay showed that when the expression of SRI was upregulated, the protein levels of STAT3 also increased significantly. At same time, anti-apoptotic proteins Bcl-2, Bcl-XL, MCL-1 and cIAP1 increased, and pro-apoptotic proteins Bax and caspase3 decreased. In contrast, when the SRI was downregulated, the expression of STAT3 decreased significantly, and the anti-apoptotic proteins Bcl-2, Bcl-XL, MCL-1 and cIAP1 decreased, while pro-apoptotic proteins Bax and caspase3 increased. Moreover, phosphorylation of p65 and IκB also increased in upSRI groups and conversely decreased in shSRI groups (Figure 4E–H).

To further verify the effect of SRI on STAT3 expression and the molecular mechanism regarding cell apoptosis in vivo, we checked tissue proteins by Western blot and immunohistochemical staining, which were consistent with the results in vitro (Figure 4I–L). Collectively, these results demonstrated that the interaction between SRI and STAT3 inhibits apoptosis and activates p65 and IκB phosphorylation, triggering the NF-κB signaling pathway in vitro and in vivo.

### 2.5. SRI and STAT3 Interaction Is Crucial for HCC Anti-Apoptosis

**Figure 5:**
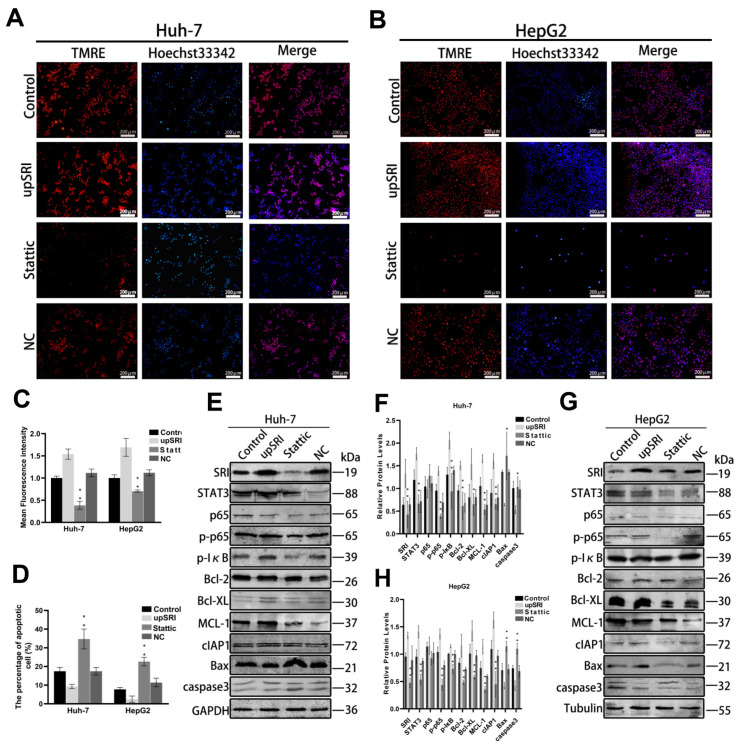
SRI and STAT3 interaction is crucial for anti-apoptosis. (**A**–**D**) Stattic reduced the fluorescence intensity of TMRE (shown in red) and enhanced the sensitivities to SRI induced apoptosis by Hoechst 33342 (shown in blue) staining assay. Scale bars = 200 μm. (**E**–**H**) The expression of SRI, p65, p-p65, p-IκB and apoptosis-related proteins were detected by Western blot in Stattic group. * *p* < 0.05, ** *p* < 0.01.

To estimate the vital role of SRI binding to STAT3 in anti-apoptosis, we inhibited STAT3 expression by inhibitor Stattic and then observed apoptosis through the MMP assay and Hoechst 33342 staining assay. Compared with the control cells and NC cells, after treatment with Stattic, the fluorescence intensity of TMRE noticeably decreased, and the number of apoptotic cells with condensed and fragmented nuclei was higher with the Hoechst 33342 staining assay (Figure 5A–D), which indicated that STAT3 is an important factor in inhibiting the apoptosis in HCC cells.

**Figure 6:**
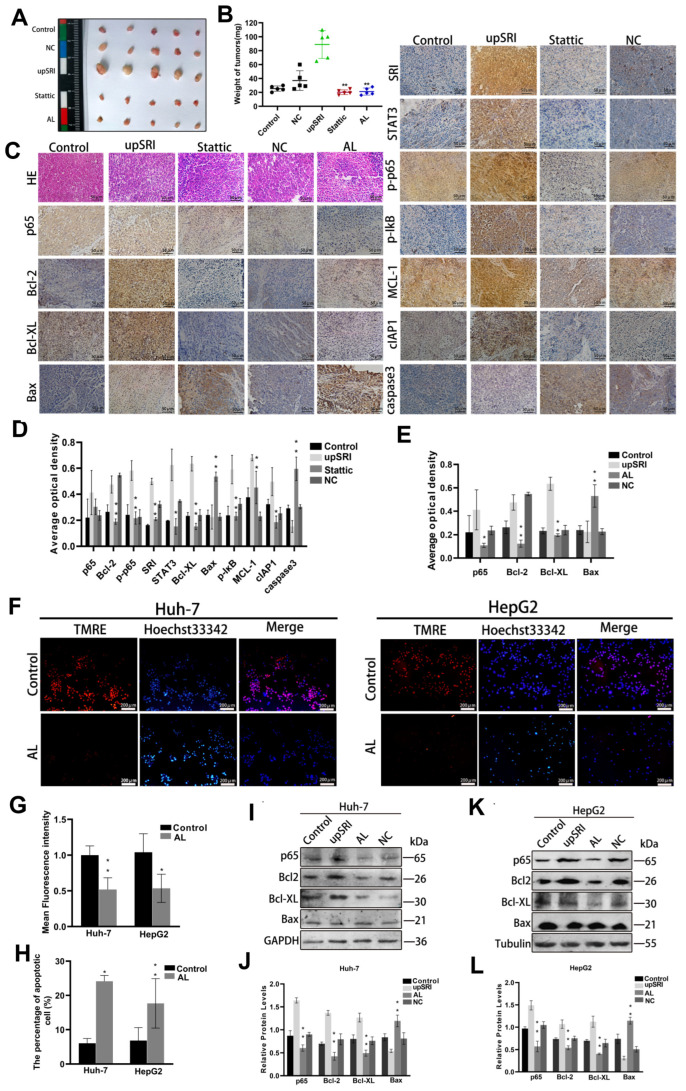
SRI inhibits cells apoptosis through the NF-κB signaling pathway. (**A**,**B**) Tumor volume and weight of orthotopic xenograft models derived from upSRI cells treated with Stattic. Symbols of different colors represented the weight of tumors in different groups. (**C**,**D**) Representative IHC images of STAT3, p65, p-p65, p-IκB and apoptosis-related proteins in Stattic treatment. Scale bars = 50 μm. (**C**,**E**) Representative IHC images of p65 and apoptosis-related proteins after treatment with AL inhibitor. The expression of proteins were determined by the brown area. (**F**–**H**) AL inhibits the fluorescence intensity of TMRE (shown in red) and reduced the sensitivities to SRI-induced apoptosis by Hoechst 33342 (shown in blue) staining assay. Scale bars = 200 μm. (**I**–**L**) Apoptosis-related proteins were detected after treated with AL inhibitor by Western blot. * *p* < 0.05, ** *p* < 0.01.

After treatment with Stattic, the expressions of p65 and IκB phosphorylation and anti-apoptotic proteins Bcl-2, Bcl-XL, MCL-1 and cIAP1 were dampened, whereas the expressions of Bax and caspase3 proteins were enhanced compared with the upSRI group (Figure 5E–H). To further evaluate the effect of STAT3 in vivo, we established a subcutaneous tumor xenograft model. The tumor weight and volume of upSRI were significantly larger and were heavier than those of the Stattic group (Figure 6A,B). The levels of p-p65, p-IκB and anti-apoptotic proteins Bcl-2, Bcl-XL, MCL-1 and cIAP1 were increased, and the levels of Bax and caspase3 were decreased in upSRI, which were attenuated in Stattic (Figure 6C,D). The results above show that STAT3 is required for SRI-induced anti-apoptosis in HCC.

### 2.6. SRI Inhibits Cells Apoptosis through the NF-κB Signaling Pathway

Regarding whether SRI inhibits apoptosis by the NF-κB signaling pathway, we treated HCC cells with Avicularin (AL), an inhibitor of NF-κB. The results of TMRE and Hoechst 33342 showed that AL significantly inhibits apoptosis (Figure 6F–H), suggesting that the NF-κB signaling pathway might stimulate the process of SRI inhibiting apoptosis. Next, in order to further identify the exact role of the NF-κB signaling pathway in SRI inhibiting the apoptosis, inhibiting AL was added to upSRI cells. As shown in Figure 6I–L, Bcl-2 and Bcl-XL were downregulated; however, Bax was upregulated in the AL group. To further strengthen our observations, we established a subcutaneous tumor xenograft model. The tumor weight and volume of upSRI were significantly larger and were further heavier in AL group (Figure 6A,B). The increase in p65, Bcl-2 and Bcl-XL was attenuated; at the same time, the level of Bax was increased in the AL group (Figure 6C,E).

## 3. Discussion

SRI, a soluble resistance-related calcium-binding protein [10,35,36,37], is often over-expressed in a number of human malignances including lung cancer, leukemia, breast cancer and gastric cancer [11,38,39,40,41,42]. Our previous work has found that SRI is overexpressed and promotes proliferation in HCC [43]. Bioinformatics analyses showed that SRI serves as a robust prognostic factor in HCC [44,45], and overexpression of SRI is associated with clinicopathological parameters, including pathological stage, T stage, OS event. Therefore, SRI could be considered as a potential molecular marker for therapeutic target of HCC, and in this work, we aimed to further explore the role of SRI in apoptosis. STAT3 is a transcription factor that has been extensively studied due to its implication in cancer. STAT3 is currently considered an oncogene, and aberrant regulation of STAT3 has been reported in many different tumors, such as glioma, thyroid cancer, breast cancer, lung cancer, osteosarcoma and so on [46,47,48,49,50]. STAT3 confers tumor great malignancy by promoting tumor invasion, migration, metastasis and angiogenesis [51], which led to it being considered a critical therapeutic target. Consistent with these finding, our data showed that STAT3 expression was also upregulated in HCC cells lines and tissues, and interacted with SRI.

Apoptosis is a regulated program of cell death that plays essential roles in tumor suppression [52]. The earliest discovered functions and primary functions of p53 are apoptosis, and p53 is well known as “guardian of the genome”. Indeed, a number of p53 targets have been identified to contribute to apoptosis [53]. In our study, we used three types of liver cancer cell lines: Huh-7, HepG2, and Hep3B. Specifically, HepG2 cells carry wild-type p53, whereas Hep3B cells have null p53, and Huh-7 cells have a point mutation at p53. However, it is not yet clear whether SRI-mediated cell apoptosis is associated with p53, which needs further investigation. The intrinsic mitochondrial apoptotic pathway is the most common form in tumors and contributes to anti-tumor therapies [54]. The Bcl-2 family of anti-apoptotic proteins are critical upstream regulators of the mitochondrial apoptotic pathway; for example, MCL-1 and Bcl-XL block cell death and only bind to apoptosis-promoting Bcl-2 effectors, such as Bax, Bak. By isolating these proteins on the outer membrane of mitochondria, MCL-1 effectively blocks MOMP, which is a necessary event in the apoptotic internal pathway [55,56]. Silencing of SRI triggers cells apoptosis through the mitochondrial pathway [11]. Downregulation of SRI promotes apoptosis in breast cancer and leukemia [41], while overexpression of SRI increases the resistance to apoptosis in leukemia K562 cells. These studies provide evidence that SRI plays a positive role in apoptosis. Therefore, we used the MMP method to detect the effect of SRI on the apoptosis of HCC cells, and found that the upregulation of SRI inhibits HCC cells’ apoptosis, while the downregulation of SRI promotes HCC cells’ apoptosis. Interesting, we found SRI and STAT3 are interacting proteins, which was analyzed by bioinformatics and confirmed by Co-IP. Protein–protein interactions play an important role in cell apoptosis [57]. In recent years, PPI has received more and more attention and become an attractive target [58,59]. Moreover, STAT3 has also been reported to be related to tumor and take part in anti-apoptosis. Therefore, the interaction between SRI and STAT3 has great potential as a new target for inducing apoptosis in HCC.

Accumulating evidence shows that the NF-κB signal pathway is involved in the pathogenesis and progression in HCC. For example, the NF-κB pathway can upregulate the expression of alpha-fetoprotein (AFP), which is regarded as a diagnostic and prognostic biomarker and a potential therapeutic target for HCC [60], and the NF-κB pathway has also been found to participate in the regulation of malignant phenotype of tumor cells [61]. The NF-κB signal pathway plays an important role in promoting the stem cell apoptosis of HCC cells [62]. Apoptosis inhibitor proteins (IAPs) are a group of anti-apoptotic proteins, including cellular-IAP1 (cIAP1) and cellular-IAP2 (cIAP2) [63]. It has been proven that cIAP1 and cIAP2 are key regulators of NF-kB signal transduction [64]. In addition, cIAP1 or cIAP2 inhibits TNFα by the NF-κB signal pathway to regulate its anti-apoptotic activity [65]. In our study, the interaction between SRI and STAT3 inhibits the apoptosis of HCC by activating the NF-κB signaling pathway in vitro and in vivo. When upregulation of SRI was found, the protein levels of STAT3 also significantly increased and p65 was phosphorylated and IκB increased, which activated the NF-κB signaling pathway; at same time, anti-apoptotic proteins (Bcl-2, Bcl-XL, MCL-1 and cIAP1) increased, and apoptotic proteins (Bax and caspase3) decreased. With the downregulation of SRI, the expression of STAT3 also decreased, and the phosphorylated p65 and IκB, apoptotic proteins and anti-apoptotic proteins also changed in the opposite direction. Collectively, SRI regulates the expression of STAT3, inhibits apoptosis and activates the NF-κB signaling pathway.

STAT3 is largely believed to be a key oncogene, and intensive efforts have been devoted to developing STAT3 inhibitors. Stattic has been shown to induce apoptosis in STAT3-dependent cancer cell lines, which selectively inhibits the activation, dimerization and nuclear translocation of STAT3 [66]. In our study, we used Stattic to further confirm the role of STAT3 in apoptosis, and found that STAT3 promoted apoptosis through the NF-κB signaling pathway in HCC.

AL quercetin-3-α-L-arabinofuranoside belongs to a group of flavonoid glycosides, and it is an inhibitor of NF-κB p65 [67]. In order to further confirm that the NF-κB signal pathway plays an important role during inhibition of cell apoptosis by SRI, we added AL and then cell apoptosis was observed. The results showed that AL significantly inhibited NF-κB p65 depending on concentration and promoted cell apoptosis, which suggested that SRI may inhibit cellular apoptosis through the NF-κB pathway.

**Figure 7:**
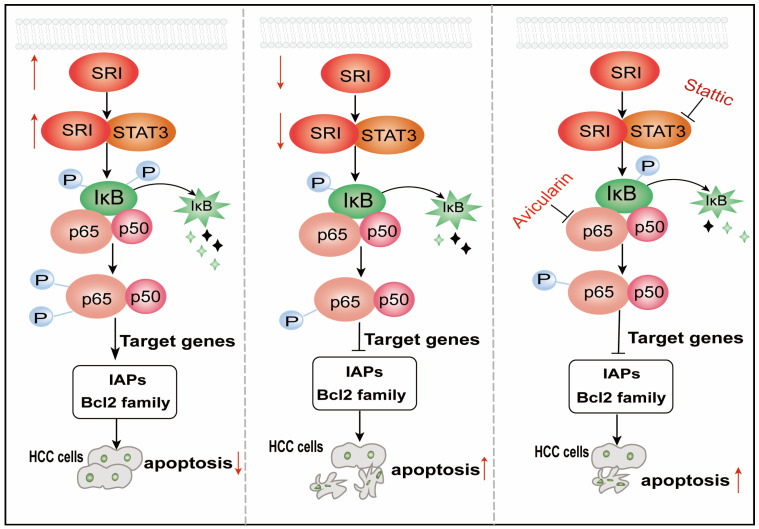
A schematic illustration of SRI regulating apoptosis in HCC. SRI overexpression inhibits the apoptosis of HCC cells by interacting with STAT3 via NF-κB pathway, whereas opposing effects were observed for knockdown of SRI. Stattic, an inhibitor of STAT3, promotes apoptosis of HCC cells via NF-κB pathway. Avicularin, the inhibitor of NF-κB, which promotes apoptosis of HCC cells. ↑: represent upregulation or promotion. ↓: represent downregulation or inhibition.

Our study unveiled a mechanism about SRI as a tumor promotor, interacting with STAT3, inhibited mitochondrial apoptosis by the NF-κB pathway in HCC (Figure 7), which suggests that SRI maybe considered as a promising therapeutic target for HCC.

## 4. Materials and Methods

### 4.1. Bioinformatics Analysis

The HCC Gene data were obtained from Gene Expression Omnibus (GEO) Profiles. The expression levels of SRI and STAT3 were analyzed in normal liver tissues and HCC tissues in GSE4882. For the correlation between SRI and STAT3, the data were from The Cancer Genome Atlas (TCGA). Clinical data for correlation analysis of SRI expression and clinicopathological features were also downloaded from the TCGA database. The potential protein–protein interaction (PPI) possibility was predicted by the STRING database. The IHC results of the proteins in HCC were from the Proteinatlas database. Scale bars = 200 μm.

### 4.2. Cell Culture

Human hepatocytes (HL-7702) and HCC cell lines (Huh-7/HepG2/Hep3B) were obtained from the Cell Bank of Chinese Academy of Sciences. The HCC cell lines (Huh-7/HepG2/Hep3B) were cultured in high Dulbecco’s Modified Eagle’s medium (DMEM) (HyClone, Logan, UT, USA), which were supplemented with 10% fetal bovine serum (FBS) at 37 °C and 5% CO_2_. Human hepatocytes (HL-7702) were routinely grown and maintained in RPMI 1640 medium with 10% FBS at 37 °C in a humidified incubator with 5% CO_2_.

### 4.3. Tissue Samples from HCC Patients

Thirty HCC samples and para-cancerous tissues samples were collected from the Dalian Public Health Clinical Center (Dalian, China). The patients included 22 males and 8 females, 15 patients ≥ 60 years and 15 patients < 60 years. 

### 4.4. Recombinant Lentivirus

The SRI full-length plasmid, shSRI (5′-GCCCTGACAACAATGGGATTT-3′) and a non-targeting sequence (negative control, NC) were synthesized by GenePharma Co. Ltd. (Shanghai, China). Huh-7/HepG2 cells (2 × 10^5^cells/well) were plated into a six-well plate, transfected with 5 μg lentivirus vectors and 1 μL Polybrene using GenePharmaTM recombinant lentivirus reagent, and then screened by puromycin (2 μg/mL, Solarbio, Beijing, China) for 2 weeks.

### 4.5. Western Blot Assay

Frozen tissues (30 mg) were grossly minced and digested in 200 μL RIPA buffer (Solarbio, Beijing, China) with 0.2 mM PMSF for 20 min on ice. Cells were collected from 10 cm dishes, washed with PBS and lysed with RIPA buffer and PMSF for 20 min on ice. The lysate was centrifuged (12972× *g*-force, 15 min, 4 °C) and the supernatant was collected.

The protein concentrations of the lysates were measured using a BCA protein assay kit (Beyotime, Shanghai, China). Equal amounts of total proteins were separated by 10% or 12% dodecyl sulfate polyacrylamide gel electrophoresis (SDS-PAGE) and transferred to polyinylidene fluoride (PVDF) membranes (Merck KGaA, Darmstadt, Germany). Membranes were blocked with 5% nonfat milk in phosphate-buffered saline tween 20 (PBST) for 1 h at room temperature. The diluted primary antibodies against SRI (1:500, Proteintech, Wuhan, China), STAT3 (1:500, Proteintech, Wuhan, China), Bcl-XL (1:500, Proteintech, Wuhan, China), MCL-1 (1:500, Proteintech, Wuhan, China), p65 (1:500, Proteintech, Wuhan, China), p-p65 (1:500, Abmart, Shanghai, China), Bcl-2 (1:1000, Abcam, Cambridge, UK), Bax (1:1000, Proteintech, Wuhan, China), caspase3 (1:500, Proteintech, Wuhan, China), cIAP1 (1:500, Proteintech, Wuhan, China), p-IκB (1:500, Proteintech, Wuhan, China), tubulin (1:5000, Proteintech, Wuhan, China) and GAPDH (1:10000, Proteintech, Wuhan, China) were incubated with the membranes at 4 °C overnight. Then, the membranes were incubated with HRP-labeled secondary antibody for 1 h at 37 °C. Immunoblot quantification was performed using Odyssey imaging system (Li-Cor Biosciences, Lincoln, NE, USA).

### 4.6. Immunohistochemistry (IHC) Assays

Firstly, the samples were fixed with 10% formalin and embedded in paraffin, and then 3 μm of blocky paraffin sections were cut and mounted into slides. Histological examination was performed with HE staining. The paraffin slices were dewaxed by xylene for 30 min and rehydrated by gradient alcohol for 5 min, respectively. Next, endogenous peroxidases were blocked with 3% H_2_O_2_ for 30 min, and incubated overnight with primary antibodies including SRI (1:100), STAT3 (1:100), Bcl-XL (1:100), MCL-1 (1:100), p65 (1:100), p-p65 (1:100), Bcl-2 (1:150), Bax (1:100), caspase3 (1:100), cIAP1 (1:100), p-IκB (1:100). The next day, the slides were washed and incubated with secondary antibody for 30 min. After washing, detection was determined by a Non-Biotin Horseradish Peroxidase Detection System and DAB substrate, then the slices were counterstained by hematoxylin. Immunohistochemistry results were observed and evaluated by pathologists. Scale bars = 50 μm.

### 4.7. Co-Immunoprecipitation (Co-IP) Assays

The cells were collected from 10 cm dishes, and then lysed in IP lysis buffer. The cell lysate was immunoprecipitated with primary antibodies overnight at 4 °C and then the immunocomplexes were collected by the addition of protein G-Sepharose beads for 3 h at 4 °C and washed with lysis buffer. The supernatant obtained after centrifugation was saved for protein detection according to the manufacturer’s protocols (IP Kit, PTGLAB, Proteintech, Wuhan, China). The proteins were followed by electrophoresis and identification by Western blot.

### 4.8. Mitochondrial Membrane Potential (MMP) Assay

Mitochondrial Membrane Potential Assay Kit (Beyotime, Shanghai, China) is a rapid detection kit for changes in mitochondrial membrane potential in cells using TMRE as a fluorescent probe. TMRE is a potentiometric, cell-permeable fluorescent indicator that accumulates in the highly negatively charged interior of mitochondria. When cells undergo apoptosis, the mitochondrial membrane potential collapses, mitochondrial permeability transition pore alters, TMRE is released into the cytoplasm, and the orange red fluorescence intensity inside mitochondria drops dramatically. In this way, changes in the mitochondrial membrane potential could be detected based on the changes in the fluorescent color. The cells were washed with PBS and then incubated with TMRE staining solution for 30 min at 37 °C in accordance with the manufacturer’s instructions.

### 4.9. Hoechst 33342 Assay

Cells at a density of 1 × 10^5^ were incubated overnight in 6-well plates. The cells were washed twice with PBS and then stained with Hoechst 33342 solution in darkness for 15 min (Solarbio, Beijing, China). The morphological changes in the nucleus were observed by fluorescence microscope.

### 4.10. Immunofluorescence Assay

Cells were fixed with 4% formaldehyde for 15 min, washed with PBS three times and treated with 0.2% Triton X-100 (Solarbio, Beijing, China) for 15 min. Next, cells were blocked with blocking solution containing 3% BSA in PBS for 15 min. SRI (1:100) and STAT3 (1:100) primary antibodies were incubated overnight at 4 °C. After washing with PBS three times, the cells were incubated with goat anti-mouse Alexa 488 and goat anti-rabbit Alexa 564 (1:200) at 37 °C for 2 h. Then, nuclei were stained with DAPI (Beyotime, Shanghai, China). Each image was examined with inverted fluorescence microscope (Olympus CKX41, Tokyo, Japan).

### 4.11. Mouse Xenograft Study

BALB/c Female nude mice, 4–6 weeks old (Vital River, Beijing, China) were maintained in the specific pathogen-free laboratory animal center. The upSRI group, Stattic group and AL group originally generated in HepG2 cells (1.5 × 10^6^) were subcutaneously injected into the neck and back of the mice. Tumor growth was carefully observed and the mice were euthanized on the 28th day after the injection of tumor cells, then the tumors were harvested and weighed. Subsequently, the tumors were dehydrated, embedded in paraffin, and sliced for HE and immunohistochemical staining.

### 4.12. Statistical Analysis

Statistical analysis was performed with GraphPad Prism 8.0. Differences between two groups were assessed by Student’s *t*-test. Intergroup comparisons were performed using a one-way analysis of variance. *p* < 0.05 was considered statistically significant.

## Figures and Tables

**Table 1:** Correlation analysis of SRI expression and clinicopathological features in HCC patients.

Characteristic	Low Expression of SRI	High Expression of SRI	*p*
T stage			0.013
T1	104 (28.3%)	77 (20.9%)	
T2	37 (10.1%)	57 (15.5%)	
T3	34 (9.2%)	46 (12.5%)	
T4	8 (2.2%)	5 (1.4%)	
N stage			0.368
N0	128 (50%)	124 (48.4%)	
N1	1 (0.4%)	3 (1.2%)	
M stage			0.622
M0	133 (49.3%)	133 (49.3%)	
M1	3 (1.1%)	1 (0.4%)	
Pathologic stage			0.025
Stage I	98 (28.2%)	73 (21%)	
Stage II	37 (10.7%)	49 (14.1%)	
Stage III	36 (10.4%)	49 (14.1%)	
Stage IV	4 (1.2%)	1 (0.3%)	
Tumor status			0.018
Tumor-free	112 (31.8%)	89 (25.3%)	
With tumor	64 (18.2%)	87 (24.7%)	
Gender			0.53
Female	57 (15.4%)	64 (17.3%)	
Male	128 (34.5%)	122 (32.9%)	
Age, n (%)			1
≤60	89 (24.1%)	88 (23.8%)	
>60	96 (25.9%)	97 (26.2%)	
OS event			0.042
Alive	130 (35%)	111 (29.9%)	
Dead	55 (14.8%)	75 (20.2%)	
DSS event			0.194
Alive	148 (40.8%)	136 (37.5%)	
Dead	34 (9.4%)	45 (12.4%)	
Age, median (IQR)	61 (51, 69)	61 (52, 69)	0.84

## Data Availability

The data that support the findings of this study are available from the corresponding author upon reasonable request.
